# A Neural Circuit Covarying with Social Hierarchy in Macaques

**DOI:** 10.1371/journal.pbio.1001940

**Published:** 2014-09-02

**Authors:** MaryAnn P. Noonan, Jerome Sallet, Rogier B. Mars, Franz X. Neubert, Jill X. O'Reilly, Jesper L. Andersson, Anna S. Mitchell, Andrew H. Bell, Karla L. Miller, Matthew F. S. Rushworth

**Affiliations:** 1 Department of Experimental Psychology, University of Oxford, Oxford, United Kingdom; 2 The Oxford Centre for Functional MRI of the Brain, Nuffield Department of Clinical Neurosciences, University of Oxford, Oxford, United Kingdom; 3 MRC Cognition and Brain Sciences Unit, Cambridge, United Kingdom; Duke University Medical Center, United States of America

## Abstract

Despite widespread interest in social dominance, little is known of its neural correlates in primates. We hypothesized that social status in primates might be related to individual variation in subcortical brain regions implicated in other aspects of social and emotional behavior in other mammals. To examine this possibility we used magnetic resonance imaging (MRI), which affords the taking of quantitative measurements noninvasively, both of brain structure and of brain function, across many regions simultaneously. We carried out a series of tests of structural and functional MRI (fMRI) data in 25 group-living macaques. First, a deformation-based morphometric (DBM) approach was used to show that gray matter in the amygdala, brainstem in the vicinity of the raphe nucleus, and reticular formation, hypothalamus, and septum/striatum of the left hemisphere was correlated with social status. Second, similar correlations were found in the same areas in the other hemisphere. Third, similar correlations were found in a second data set acquired several months later from a subset of the same animals. Fourth, the strength of coupling between fMRI-measured activity in the same areas was correlated with social status. The network of subcortical areas, however, had no relationship with the sizes of individuals' social networks, suggesting the areas had a simple and direct relationship with social status. By contrast a second circuit in cortex, comprising the midsuperior temporal sulcus and anterior and dorsal prefrontal cortex, covaried with both individuals' social statuses and the social network sizes they experienced. This cortical circuit may be linked to the social cognitive processes that are taxed by life in more complex social networks and that must also be used if an animal is to achieve a high social status.

## Abstract 2

A neural circuit that covaries with social hierarchy A neuroimaging study reveals that individual variation in brain circuits in structures below the cerebral cortex of macaques is associated with experience at different ends of the social hierarchy.

## Author Summary

Social status is an important feature of group life in many primates. Position in the dominance hierarchy influences access to food and mates and is correlated with both general and mental health. Discovering how the brain is organized with respect to individual social status is an important first step for understanding the neural mechanisms that might drive social status and mediate its consequences. We performed a neuroimaging study in non-human primates and our findings suggest that brain organization reflects at least two aspects of dominance. First, we identified neural circuits in brain regions that appear to have a relatively simple and direct relationship with social status—one circuit in which gray matter volume tended to be greater in socially dominant individuals and another in which gray matter volume was greater in those with a more subordinate social position. We also showed that the degree of connectivity within each circuit was associated with experiences at each end of the social hierarchy. Second, given that social status in male macaques depends not only on successful engagement in agonistic behavior but also on success in forming social bonds that promote coalitions, we explored regions where gray matter relates to both social status and social network size. This second neural circuit may mediate the way in which dominance is dependent on social bond formation, which is in turn dependent on social cognition.

## Introduction

Social status is a salient feature of group life in many primates including humans and macaques [1]. Position in the dominance hierarchy influences access to food and mates and is a predictor of health and reproductive success [2]. It is also associated with individual differences in behavior; for instance, more dominant macaques make more prosocial choices and are more likely to make a choice that leads to reward for both themselves and another as opposed to a choice that leads just to reward for themselves [3],[4]. Although subordinate animals pay attention to social cues provided by animals from any social rank, dominant animals follow information provided by other dominant animals [5].

Despite widespread interest [6],[7] the neural correlates of social status in primates are largely unknown. Identifying brain areas in which structure and function are related to social status is an important first step for understanding the neural mechanisms that might drive social status and mediate its consequences. Serotonin has been linked to dominance status; pharmacological manipulations that increase or decrease serotonergic activity lead, respectively, to increases and decreases in dominance status in monkeys [8]. However, the wider neural system in which serotonin operates in relation to dominance in primates has proved difficult to investigate.

An alternative approach to studying the neural correlates of primate dominance has been to induce relative differences in social status during the playing of interactive games in human subjects. The amygdala was recently implicated in tracking social hierarchy during performance of such a game in human subjects [9], but the degree to which playing brief and artificial games induces or simulates the often protracted differences in dominance status experienced by primates, including humans, in the real world remains unclear.

Here, we investigate the neural correlates of social status by relating spontaneously occurring social dominance status in 25 captive macaques to differences in their brains' gray matter (GM), measured with structural MRI, and the spontaneous coupling of activity between brain regions, measured with functional MRI (fMRI). Quite distinct aspects of macaque behavior have been related to neural structure and function in this way in the past [10]. MRI has the advantage of furnishing quantitative measurements of neural data across the whole brain. However, some of the neural structures implicated in other fundamental aspects of emotional and motivational behavior are subcortical and small in size and so they are difficult to investigate with non-invasive approaches.

Questions remain, however, over the best methods to ensure the reliability and robustness of whole brain GM differences. The originators of MRI voxel-based GM analyses emphasized that taking into account the spatial extent, across adjacent MRI voxels, of any statistical effect may not be appropriate in GM analyses [11]. Instead they suggested an alternative approach for testing whether or not relationships between GM and behavioral or disease variables are robust; they suggested examining whether effects are bilaterally symmetrical [12]. The approach of finding similar effects in bilaterally symmetrical structures was used in some of the earliest human GM analyses by some of these investigators [13] and has continued to be used [14]. It rests on the assumption that if a statistical effect noted had a chance of occurrence of *p*<0.001 in one brain area under the null hypothesis, then it has the chance of occurring in the same area in both hemispheres with the square of this probability (i.e., *p*<0.00001) [12],[14]. The convention adopted in these studies in recent years has been to focus on effects that extend over 10 voxels [14] and so we have adopted the same approach in our study.

In addition to the whole brain approach, we also adopted a hypothesis-driven region of interest (ROI) approach widely used in human neuroimaging. We examine effects in predefined ROIs using a threshold of *p*<0.05 and correction for multiple comparisons across all voxels in the ROI. As noted above, there are *a priori* reasons for thinking that dominance may be associated with the amygdala and the serotonergic system. We cannot, with a non-invasive approach such as MRI, selectively examine neurons identified as serotonergic, but we can examine whether the brainstem nuclei containing many serotonergic nuclei have any association with dominance. As explained below, the two analysis approaches, the ROI-led and the more exploratory search for bilaterally symmetric effects, suggest similar conclusions.

In addition to social status, another major aspect of social life is overall size of the social network that an animal experiences. Previously we reported that structure and activity in a specific network of cortical areas in the macaque, centered on the mid-superior temporal sulcus (mSTS), anterior cingulate cortex (ACC), and dorsal and rostral prefrontal cortex (PFC) reflects each macaque's social group size [15]. In an additional analysis, we contrasted the relationship between brain structure and these two social variables: social status and social network size. We identified subcortical regions that were solely related to social status and cortical regions linked to social status and social network size. The pattern of results suggested the existence of two distinct networks related to different aspects of social status.

## Results

### Relationship between GM and Social Status in ROI-Based Analyses

We obtained structural and fMRI scans of 25 macaques living in groups of five, four, three, and two individuals (five, twelve, one, and seven animals in each case) after assigning each animal a cardinal index of dominance on the basis of its behavioral interactions (based on [16]). Deformation-based morphometric (DBM) analysis was used to identify regions in the left hemisphere where GM covaried with social status. This analysis specifies the expansion/contraction required at each voxel in the group average templates scan to deform the brain to an individuals' brain. A discussion of these types of methods can be seen in [17].

**Figure 1:**
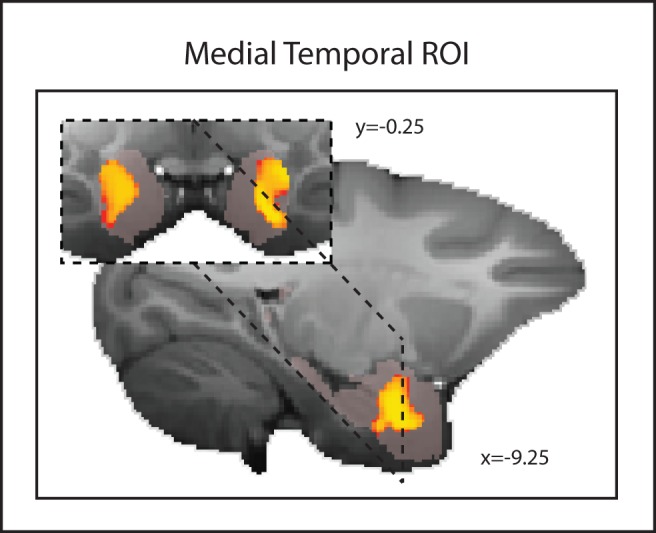
Areas of GM significantly (*p*<0.05 corrected for multiple comparisons) related to social status in an ROI covering the left and right amygdala. The medial temporal ROI mask is shown in translucent pink.

First, we report the results of tests examining the relationship between dominance and GM, after controlling for age, weight, sex, and number of structural scans included in the DBM (see Methods) [15], in a 2752.5 mm^3^ (22,020 voxels) ROI (Figure 1) centered over bilateral medial temporal lobe so as to include the whole amygdala. After correcting for multiple comparisons across all voxels in the ROI, we were able to identify voxels extending from the central AMY to the border with the hippocampus in both left and right hemispheres that showed a relationship with dominance (*p*<0.05 corrected for multiple comparison; Figure 1).

**Figure 2:**
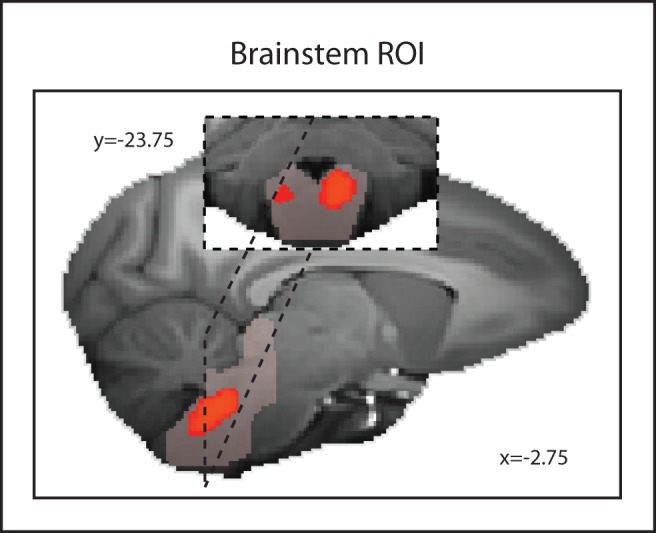
Areas of GM significantly (*p*<0.05 corrected for multiple comparisons) related to social status in an ROI covering the brainstem including raphe nucleus and adjacent reticular formation. The brainstem ROI mask is shown in translucent pink.

Next we examined a 2,574.5 mm^3^ (20,596 voxels) bilateral ROI (Figure 2) in the brainstem between the medulla and the midbrain so as to include the location of the serotonergic nuclei. Using the same approach in the amydala investigation, we identified voxels both to the left and right of the midline with a significant association with dominance (*p*<0.05 corrected for multiple comparisons; Figure 2). The region included parts of the RN and the adjacent reticular formation such as the gigantocellular reticular nuclei.

### Subcortical Areas Linked to Social Status in the Left Hemisphere

**Figure 3:**
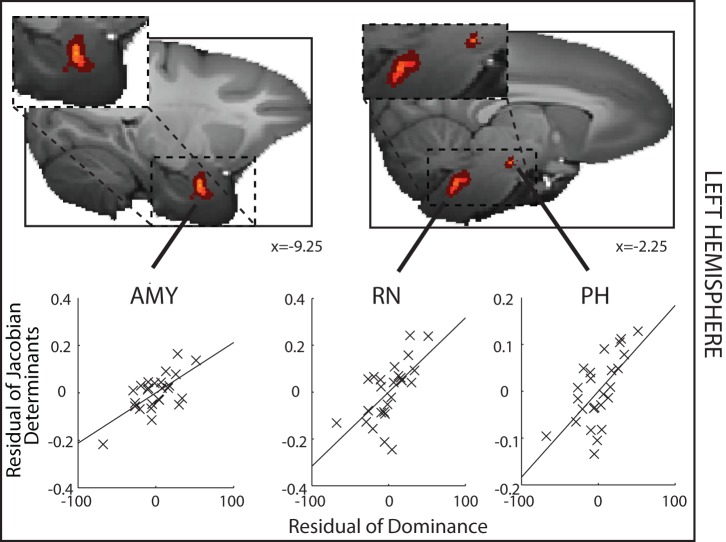
Left hemisphere DBM results where GM is significantly related to social status. A significant positive correlation between GM and social status—dominance—is found in the AMY, PH, and adjacent lateral hypothalamus and RN and adjacent reticular formation. The determinant of the Jacobian matrix summarizes the size of the GM effect. For illustration purposes, partial correlations between the determinants of the Jacobian for each animal and for each ROI and dominance, after controlling for age, weight, sex, and number of structural scans, are shown. Spatially uncorrected effects (*p*<0.001, volume>1.25 mm^3^) are indicated by light (yellow) colors, whereas the darkest red color indicates effects at *p*<0.005 (cluster volume>5 mm^3^).

Next we employed the approach of looking for bilaterally symmetrical brain regions in which GM exhibited similar relationships with dominance [12]–[14]. We used the same threshold as in other recent human GM investigations [14]: *p*<0.001; volume>1.25 mm^3^, equivalent to 10 adjacent voxels. The chance that bilaterally symmetrical effects would be found in the two hemispheres under the null hypothesis is, of course, even lower (*p*<0.00001). In order to explain the approach, we first report how we sought regions of GM in the left hemisphere where the deformation was significantly related to social status after controlling for age, weight, sex, and number of structural scans included in the DBM (see Methods) [15] and identified (1) the AMY, (2) a region centered on the posterior hypothalamus (PH) but extending into adjacent lateral hypothalamus, and (3) a brainstem region that again included parts of the RN and the adjacent reticular formation such as the gigantocellular reticular nucleus (Figure 3 and Table S1). In other words, this alternative approach identifies the same two areas that were identified in the ROI approach and a third region—the PH.

**Figure 4:**
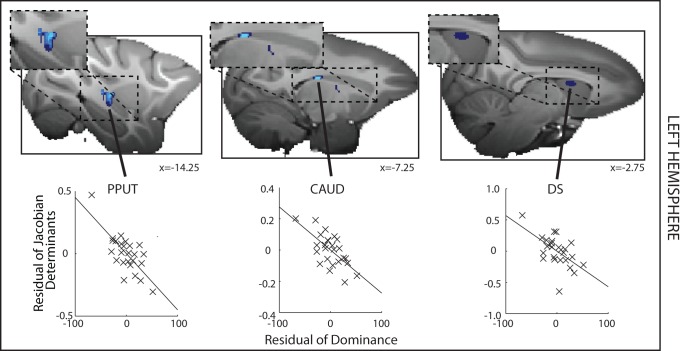
Left hemisphere DBM results where GM is significantly negatively related to social status. A significant negative correlation between GM and social status—subordinance—is found in the PPUT and tail of the CAUD. Note the dorsal striatum and dorsal septum (DS) appears only at the more liberal threshold of *p*<0.005, cluster volume>5 mm^3^. Format, colors, and methods are the same as in Figure 3, but now stronger effects (*p*<0.001, volume>1.25 mm^3^) are shown in light blue and weaker effects in dark blue (*p*<0.005, cluster volume>5 mm^3^).

We also sought regions of GM that significantly correlated with decreased dominance, or subordinance scores. We identified several regions within the striatum and the adjacent septum. These comprised the posterior putamen (PPUT), and the tail of the caudate (CAUD) (Figure 4 and Table S1). Using a slightly more liberal statistical threshold of *p*<0.005 (volume>5 mm^3^), however, we were also able to identify a relatively extensive region in the anterior dorsal striatum, in the caudate nucleus, that extended into dorsal parts of the septum (DS) (Figure 4). We note the existence of this region here because we again found evidence for a negative relationship between all three of these brain structures and dominance even at the more stringent statistical criterion level of *p*<0.001 in the right hemisphere (see below).

### Subcortical Areas Linked to Social Status in the Right Hemisphere

**Figure 5:**
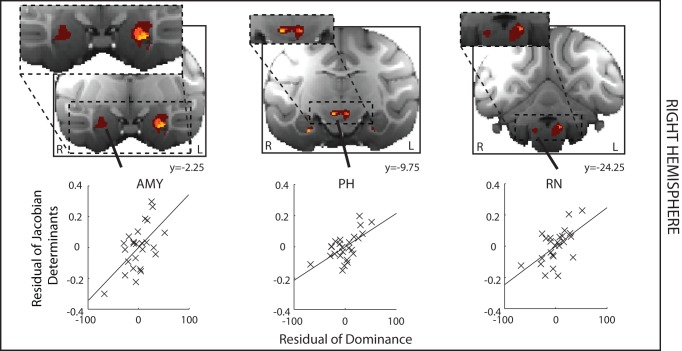
DBM results where GM is significantly related to increased social status in the right hemisphere (shown on the left of the figure). The areas identified include AMY and PH (*p*<0.001, volume>1.25 mm^3^) and an extensive brainstem region (RN; *p*<0.005, volume>5 mm^3^). There is a clear bilateral symmetry in the positions of effects. Format, colors, and methods are the same as in Figure 3.

**Figure 6:**
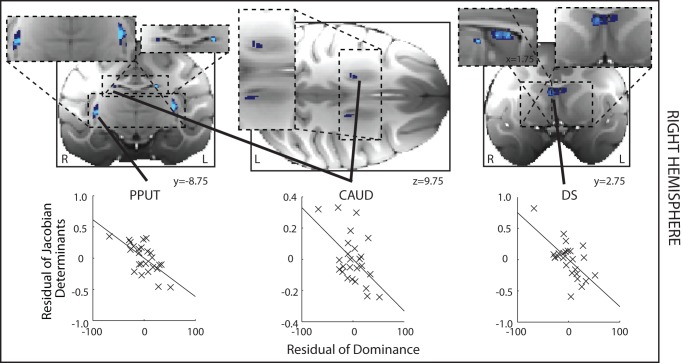
DBM results where GM is significantly related to decreased social status in the right hemisphere (shown on the left hand side of the figure). The areas identified include PPUT, CAUD, and DS. There is a clear bilateral symmetry in the positions of effects. Format, colors, and methods are the same as in Figure 3.

To examine the reliability of the effects we had found in the left hemisphere, we next examined whether effects could be found in the other hemisphere. A similar approach has been used in human neuroimaging experiments [12]–[14]. We sought regions of GM in the right hemisphere where the deformation was significantly related to social status (positive and negative correlations are shown in Figures 5 and 6, respectively). Notably all regions survived at our strigent criterion (*p*<0.001, volume>1.25 mm^3^), with the exception of the RN, which survived at *p*<0.005 (cluster volume, 6.5 mm^3^). Importantly, at this threshold, the effect was spatially extensive and included many voxels at which the effect was significant at *p*<0.001 (Figure 5, righthand panel).

Again in the right hemisphere, as in the left hemisphere, we also found evidence of a negative relationship between GM and social status in PPUT, CAUD, and DS. Notably the DS region that had been tentatively idenfied in the left hemisphere was the largest cluster to survive within the analysis at *p*<0.001 of right hemisphere effects (cluster volume, 5.375 mm^3^).

On reviewing the illustrations of the partial correlations between GM and dominance in each ROI, we noted that it might be argued that the animal with the lowest dominance score was an outlier. Although this animal's dominance score was within two standard deviations of the mean, we nevertheless checked whether the relationship between dominance and GM in each ROI still held after excluding this individual. We found that this was indeed the case, with the exception of the right CAUD ROI, where the correlation remained only marginally significant (*p* = 0.065). In order to further corroborate the link between GM in the ROIs, we collected a second set of dominance scores and carried out a second set of MRI scans of the animals. We turn to the results of this follow-up investigation next.

### Subcortical Areas Linked to Social Status: A Follow-Up Investigation

**Figure 7:**
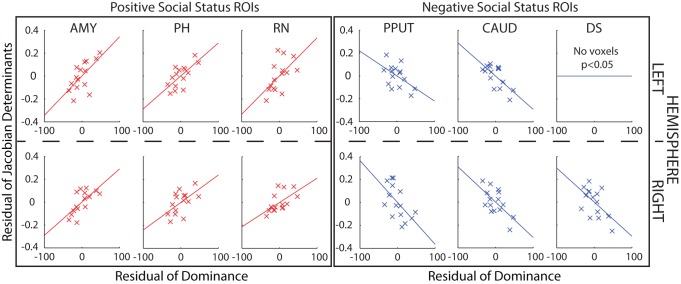
DBM effects in 15 animals at a second time point. GM in 11 of the 12 ROIs still correlated significantly with social status. For illustrative purposes, scatter plots of the Jacobian determinant extracted from a 3.375 mm^3^ mask placed over the peak coordinate for each contrast (uncorrected *p*<0.05) against dominance are shown after controlling for age, weight, sex, and number of structural scans.

After an interval of 4 to 5 months we were able to collect a second structural scan as well as a second independent measure of dominance for a homogeneous subgroup of 15 monkeys drawn from the original group of 25. We focused on male macaques because nearly all the macaques in the initial investigation had been male macaques and living in groups of four or five individuals (see Table S3), but otherwise we attempted to include as many of the original animals as possible. Because the animals were assigned to other neuroscientific experiments, it was only possible to reinvestigate 15 monkeys at this second time point. This second measure of dominance confirmed that, despite some changes in group membership (two of the monkeys previously housed in a group of four were now housed in a group of two), social hierarchies were relatively stable across the time spanned by the two assessment periods; there was a correlation between the social statuses assigned to the 15 monkeys measured on the two different occasions (r = 0.681, *p* = 0.005). We therefore examined whether the same relationship between brain structure and dominance could be detected in this new data set. Instead of running whole brain analyses in what was now a smaller group of animals, we focused on the six bilateral ROIs identified from the first experiment. Twelve 166.375 mm^3^ (equivalent to 11 voxels^3^ at a resolution of 0.5 mm) ROI masks were positioned over the coordinates calculated as the centers of gravity of the clusters identified in the previous analyses. Within these ROIs, even after controlling for age and weight, we once again found positive correlations between GM and dominance in AMY, RN, and PH in both left and right hemispheres and negative correlations in CAUD and PPUT in both left and right hemispheres and in right DS. The only effect of the original 12 that was not observed on the second occasion was the negative one between left DS and social status (Figure 7 and Table S2).

### Interregional Interactions in Activity Covary with Social Status

The first three analyses examined relationships between social status and brain structure. In the next analysis, we sought to determine whether dominance was associated with differences in functional activity. We looked at activity measured in the resting state and indexed by the blood oxygen level–dependent (BOLD) signal measured with fMRI. It has previously been shown that the strength of BOLD coupling between certain cortical regions is correlated with the social network size that an individual animal experiences [15] and so, by analogy, here we measured whether the strength of activity coupling between pairs of regions identified in the first three analyses was correlated with social status.

In order to avoid making multiple comparisons of the many possible pairs of areas, we focused our analysis in two ways. First, we again focused on just the left hemisphere—the hemisphere in which we had begun our analysis and in which, on average, effects were perhaps slightly stronger. Second, we examined the correlations in resting state activity in a network of areas with a positive relationship with dominance and a network of areas with a negative relationship to dominance. Therefore, we focused on coupling between the largest area identified as having a positive relationship with dominance (AMY) and each of the other two areas that also had a positive relationship with dominance (PH and RN) and then on the largest area identified as having a negative relationship with dominance (PPUT) and the other two areas that also had a negative relationship with dominance (DS and CAUD). Note, however, that we are not testing alternative hypotheses about the coupling between just particular pairs of areas, rather than others, being related to dominance. We are testing the more general hypothesis that coupling within this network of areas is related to dominance. If such a relationship exists, then we might observe evidence for it in the coupling strengths between more than one pair of areas (i.e., that both interregional AMY–PH and AMY–RN coupling correlate with dominance).

To carry out the analysis, we first extracted the raw BOLD time series for every 3.375 mm^3^ ROI mask registered individually in each of the 25 animals. We did this after controlling for fluctuations in BOLD signal across the whole brain GM, white matter, and cerebral spinal fluid. Next, for each animal, the partial correlation coefficient between the resulting BOLD time series in each pair of areas (AMY and RN, AMY and PH, PPUT and CAUD, PPUT and DS) was calculated after controlling for all other ROI time series in both hemispheres (the 10 other areas identified in Figures 1–7—in other words, after controlling for the BOLD time series in the other left hemisphere areas and all the right hemisphere areas). This first stage of the analysis establishes the relationship between the activity levels in each pair of areas, after controlling for the effects of other areas, in each individual animal. The next stage of the analysis is then to test whether variations in the strength of these interareal coupling relationships across individuals were related to dominance.

**Figure 8:**
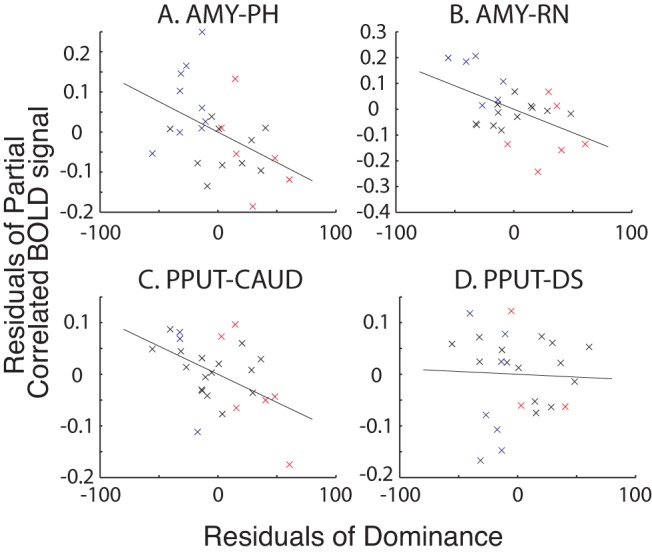
Partial correlation between interregional coupling of activity and social status after controlling for age, weight, and sex. There were significant negative correlations between AMY–PH coupling (A), AMY–RN coupling (B), and PPUT–CAUD (C) but not PPUT–DS (D) coupling and dominance. Individual dominant (red) and subdominant (blue) animals in which interregional coupling was significantly positive (in subdominant animals) and negative (in dominant animals) are also shown. All other animals, where the interregional correlation was not significant at the individual subject level, are shown in black.

In order to examine how variations in the strength of interareal coupling across individuals are related to dominance, the next analysis was conducted at the “group level”—on data from all individuals. At the group level, the resulting partial correlation values were Fisher-transformed and entered into a correlation with the residual variance of individual social status values after controlling for age, weight, and sex. This analysis indexes interareal BOLD coupling between areas in individuals and the individuals' dominance statuses. Significant negative relationships were found between dominance and the activity coupling between left AMY and PH (r = −0.423, *p* = 0.035; Figure 8A), the activity coupling between left AMY and left RN (r = −0.477, *p* = 0.016; Figure 8B), and the activity coupling between left PPUT and CAUD (r = −0.466, *p* = 0.019; Figure 8C). The relationship between dominance and PPUT–DS activity coupling, however, was not significant (r = −0.039, *p* = 0.853; Figure 8D). Statistically, under the null hypothesis that there is no real relationship of BOLD coupling between each pair of areas and dominance at *p*>0.05, the chance of our finding evidence of coupling being correlated with dominance in three of the pairs of areas is actually *p*<0.0025.

In general, a subordinate social status was associated with positive coupling between activity levels in pairs of the areas, but as social status rose, there was more likely to be negative coupling between areas. We are unable yet to explain the directions of the relationships between coupling strength and social status; however, we note that this is generally the case in human neuroimaging experiments too. Although a negative coupling between BOLD signals might indicate that one area is exerting an influence, via inhibitory interneurons, on the other area, what is important is that there is a relationship between a behavioral or cognitive variable and BOLD coupling as opposed to the specific sign of the coupling. For example, in one of the brain systems in which activity coupling changes have been most extensively investigated, the premotor–motor system, it is known that coupling between two areas changes between negative and positive within a matter of milliseconds as an individual transitions from being at rest to engaging in a behavior [18]–[24]. Whether the same is true for the subcortical brain regions we investigated here can only be determined in further experiments with awake behaving animals.

The BOLD coupling relationships are relatively strong. This is illustrated by the fact that several subdominant individual animals (animals in which dominance scores were less than the median score of 31.25) had activity coupling that was significantly positive at the individual time series level (*p*<0.01; blue crosses in Figure 8), whereas several dominant animals (animals in which dominance scores exceeded the median score of 31.25) had activity coupling that was significantly negative at the individual time series level (*p*<0.01; red crosses in Figure 8). Chi-squared tests confirmed the existence of a significant relationship between the presence of a positive coupling pattern and subdominant status and a negative coupling pattern and dominant status for the AMY–PH and AMY–RN pairs (*p* = 0.041 and *p* = 0.050, respectively).

### Contrast and Interaction of Neural Networks Correlated with Social Status and Social Network Size

We have previously reported that GM in the mSTS and rostral and dorsal PFC correlates with the sizes of macaques' social networks [15]. Animals experienced extensive contact with social networks of different sizes (of between one and seven individuals) in which they lived for most of each day. The group sizes were artificially determined; they were set by the needs of various neuroscientific experiments that would be carried out. Such group sizes are smaller than the group sizes that occur in the wild, but nevertheless they are larger than the pairwise interactions typically studied in neuroscientific investigations of social behavior in macaques [25]–[31]. In addition, an attractive feature of such group sizes is that it ensures all animals within a given group come into proximity with one another on a daily basis. More GM was found in mSTS and rostral and dorsal PFC when animals experienced larger social networks. The next question, therefore, is whether the six subcortical areas (Figures 1–6) identified in the present analysis of social status also have any relationship with social network size. The two factors, social network size and social status, should not be correlated with one another, and this was the case in the present sample (Pearson's r = −0.31, *p* = 0.126, Spearman's r = −0.24, *p* = 0.256). The lack of correlation between the two factors means that it is possible to identify variance in GM that is related to either social network size or dominance, or both.

**Figure 9:**
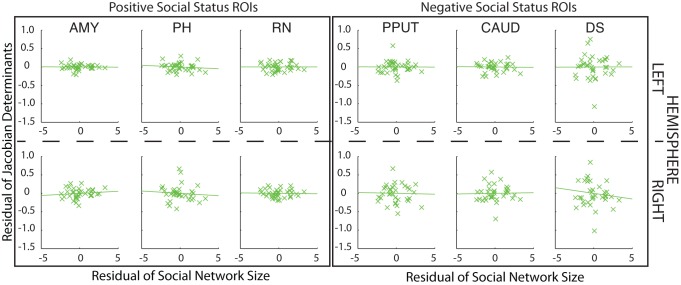
DBM effects of social network size in areas identified as having a relationship with dominance status in Figures 1–8. No significant correlation between GM and social network size was found in any ROI.

The most stringent and sensitive test for any impact of social network size on the brain areas reported in Figures 1–7 is to examine the largest number of animals for which data are available. Because we now have MRI and social network size measurements for 36 animals, we used this large group when testing whether social network size was associated with GM in the six subcortical ROIs. Note, however, that because we had not obtained dominance status measures from all of these animals, we had only been able to analyze data from 25 animals in our investigation of dominance. Despite the sensitivity of a social network size test involving 36 individuals and an ROI-based approach, no significant correlation with social network size was found even after controlling for age, weight, sex, and number of scans [left ROIs, AMY (r = −0.015, *p* = 0.932), CAUD (r = −0.021, *p* = 0.905), DS (r = 0.003, *p* = 0.987), PH (r = −0.100, *p* = 0.983), PPUT (r = −0.004, *p* = 0.983), and RN (r = 0.001, *p* = 0.997); right ROIs, AMY (r = 0.103, *p* = 0.548), CAUD (r = 0.019, *p* = 0.911), DS (r = −0.099, *p* = 0.567), PH (r = −0.072, *p* = 0.675), PPUT (r = −0.023, *p* = 0.896), and RN (r = −0.026, *p* = 0.880); Figure 9]. Further direct comparisons confirmed no differences between sexes. This suggests that individual variation in dominance status in the six subcortical regions we have identified in the present report is specific to this aspect of social behavior and unrelated to other aspects of social behavior associated with experience of social networks of different sizes. This was confirmed by showing that all of the relationships between social dominance and GM in these six regions remained significant even when social network size was partialled out from the residual GM effects (all r<−0.525 or >0.573 and all *p*<0.01). Moreover, the resting state fMRI coupling analyses that reported a link between BOLD coupling and dominance in Figure 8 remained significant even after the effect of social network size was similarly partialled out (all r<−0.385, all *p*<0.05).

**Figure 10:**
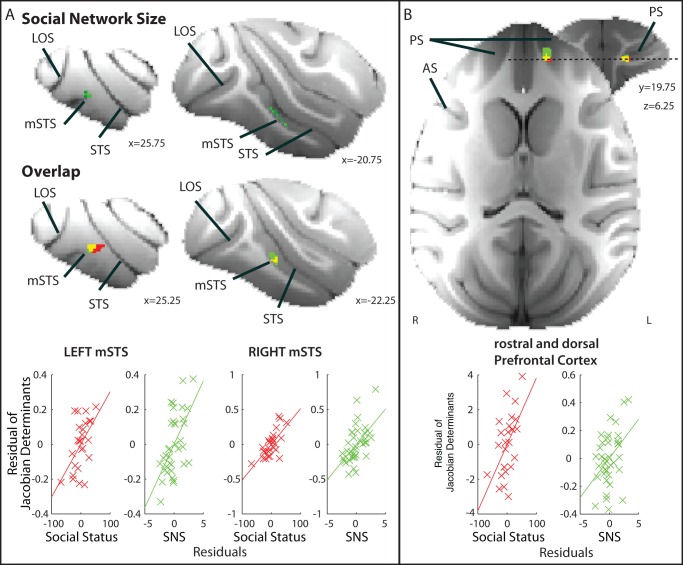
Social network size. (A) Regions of GM significantly correlated with social network size [top panel, *p*<0.001 uncorrected, bilateral cluster extent 2.625 mm^3^ (left) and 5.375 mm^3^ (right)] and overlapped with dominance status in right and left mSTS and extending onto the immediately adjacent inferior temporal gyrus (middle panel; cluster corrected *p*<0.05). For illustrative scatter plots, the Jacobian determinants from the dominance (red) and social network size (SNS; green) analyses were extracted from 3.375 mm^3^ masks placed over the centers of gravity of left and right hemisphere overlap clusters and compared with dominance (red) or social group size (green) after controlling for age, weight, sex, number of structural scans (lower panel). Voxels in which portions of the variance in GM were explained by both social network size and dominance appear as yellow. (B) Voxels in which GM is significantly correlated with social network size and with social status overlap in the rostral and dorsal PFC in the principal sulcus (right panel; cluster corrected *p*<0.05). The scatter plots are made in the same way as for (A). PS, principal sulcus; AS, arcuate sulcus; LOS, lateral occipital sulcus; STS, superior temporal sulcus.

Neural networks associated with effects of social network size and social status did, however, overlap at two points in the cortex that we have previously linked to social network size—the mSTS and rostral and dorsal PFC [15]. ROI examination based on areas identified in our previous study revealed the effects of both social status and social network size overlapped within voxels in both left and right mSTS (*p*<0.05 corrected for multiple comparisons; cluster volumes, 3.875 mm^3^ and 1.375 mm^3^ right and left, respectively; Figure 10A); even after taking into account age, weight, and sex, increased mSTS size correlated with higher social rank. In the rostral and dorsal PFC, effects of social network size and dominance were found in adjacent voxels in close proximity in the anterior part of the principal sulcus. When we tested for overlap of social network size and dominance in a small ROI between these voxels, we again found positive results (*p*<0.05 corrected for multiple comparisons; cluster volume, 1.75 mm^3^; Figure 10B).

### Hormone Metabolite Levels and Dominance

It has been suggested that some hormone levels correlate with dominance in monkeys, but the relationship is complex and influenced by numerous factors and perhaps is only clear when very large group sizes are investigated [32],[33]. We measured two hormone-related metabolites in 13 of the male macaques participating in the second study of dominance (see Table S3). We assessed fecal levels of a metabolite of cortisol, associated with stress, by measuring the 3α,11ß-dihydroxy structure cortisol metabolite (3α,11ß-dihydroxy-CM), and we assessed fecal androgen metabolite levels by measuring epiandrosterone, which reflects testicular function. Naturally, a logical step would be to test correlations between individual hormone levels and differences in structure or functional couples; however, statistical power in our sample would be too low to infer meaningful relationships.

We were unable to find a relationship between dominance and cortisol metabolite regardless of whether or not effects of social network size, age, and weight were partialled out. We did, however, find evidence for a significant relationship between epiandrosterone and dominance after partialling out effects of age, weight, and social network size (r = −0.703, *p* = 0.023). This pattern of results suggests that stress, at least as indexed by cortisol metabolite levels, is unlikely to mediate the relationship between the brain areas in Figures 1–8 and social status.

## Discussion

We identified two types of social-status–related brain regions. The evidence is particularly clear regarding the association between these areas and dominance in male macaques; all but three of the macaques in the first sample investigated were males, and all the macaques studied in the second sample were males. The first type (Figures 1–6), which have been identified, to our knowledge, for the first time in the present study comprised a network centered on (1) the amygdala (AMY) and closely interconnected structures including (2) posterior hypothalamus (PH), (3) brainstem regions including the raphe nucleus(RN) and adjacent reticular formation areas such as the gigantocellular reticular nucleus. We also identified regions of the basal ganglia—(4) the posterior putamen (PPUT), (5) caudate tail (CAUD) and (6) dorsal striatum/lateral septum (DS). It is composed of two parts in which GM was either positively (AMY, RN, PH) or negatively (PPUT, CAUD, DS) correlated with social status. In each case, the relationship with social status was a simple, direct, and strong one and spatially extensive, bilateral, and found in a series of analyses of both GM and BOLD coupling (Figures 1–8). Relationships between GM and activity coupling and social status in these subcortical areas were observed, but there was no evidence of relationship with other variables such as social network size. The second network involved cortical regions, such as mSTS and rostral PFC, where GM covaried both with social status and with social network size. We discuss this second network at the end of the Discussion section.

It is perhaps worth drawing attention to the fact that an assumption behind our analysis is that there is something about the experience of being subordinate (or conversely dominant) in a small group that is shared with the experience of being subordinate (or conversely dominant) in a larger group even if all aspects of the experience are not identical. By including a factor of dominance as well as a factor of social group size in our analyses, we identify variance in GM and brain activity that is related to the experience of being subdominant or dominant independent of the impact of group size.

As explained in the Introduction, we focused only on brain areas that exhibited a consistent relationship with social status in a series of four distinct tests. A relationship between GM in the six subcortical structures was found in both left and right hemispheres. In a further test, when structural MRI and social status measurements were repeated after a 4- to 5-month interval, we again observed similar relationships between GM in these six regions and social status. The only exception was that although the social status effect in DS was found once again in the right hemisphere, it was not found again in the left hemisphere. Furthermore, an additional test showed that the strength of coupling between fMRI-measured activity in different parts of the subcortical network changed as a function of social status.

Whether neural activity in these brain regions determines or is determined by dominance is still to be ascertained. In theory, to address this question, animals could be repeatedly assigned and reassigned from one social group to another, and an attempt could be made to carry out a longitudinal analysis of dominance effects. Although such a manipulation is theoretically possible, it would be disruptive to the animals' welfare. In our colony, in line with national guidelines, emphasis is placed on the welfare benefits of stable group housing. Another potentially interesting approach is to test whether incidental changes in dominance that occur spontaneously can be ascribed to prior neural changes. For such an approach to work, however, it is necessary for dominance changes over time to be sufficiently substantial that successive dominance measures are uncorrelated with one another. If such a change occurs, then it would be possible to test if neural measures are more predictive of later dominance than current dominance. However, sufficiently large incidental changes in dominance did not occur during our study period; dominance scores at the two time periods we studied were highly correlated (r = 0.681, *p* = 0.005). Such an approach might also require more frequent neural measurements and dominance measurements to be taken so that putative predictive neural changes were not missed.

It has been suggested that humans and monkeys assign values to themselves, “self-values,” just as they assign values to environmental stimuli [34]. Social rank is likely to be an important determinant of such self-values. Activity in some of the regions we identified, such as parts of the striatum, is known to encode many parameters that determine the values of choices [35], and our results suggest the same regions may also encode longer term valuation signals reflecting each individual's status. Recently neurons have been reported in the caudate that track a monkey's own social status during an interactive food-grab game with a competitor. These cells showed decreased activity when an individual was in a submissive state and the competitor was performing successfully and was actively taking most food items in the task [27]. It is also now clear that caudate cells encode not just the actions that an individual macaque makes but the actions made by other macaques present [26],[36].

The amygdala is also known to be important for tracking some aspects of reward value [37] and lesions to the amygdala affect socioemotional behaviors such as defense and approach tendencies [38]. The prevalence of such behaviors covaries with social status. Amygdala activity in humans has been linked to the tracking of social hierarchies during an investment game [9]. The degree to which such brief games provide insights into dominance hierarchies has been questioned, but the present results, which included particularly extensive regions of amygdala GM associated with social status, confirm the importance of the amygdala as a neural correlate of dominance.

Serotonin levels have been related to social status. Raleigh and colleagues [39] examined the consequences of removing the dominant individual from groups of vervet monkeys when serotonin levels were either increased or decreased in one of the remaining group members. Animals in which serotonin levels had been increased were more likely to accede to the dominant position than animals in which serotonin levels had been decreased. Although we did not measure serotonin directly in the current study, the brainstem regions we identified in the present study have been associated with serotonin; in the rodent, not only is the raphe nucleus a source of serotonin, but serotonergic neurons are found in other areas such as the gigantocellular raphe nucleus [40]. Such serotonergic neurons may influence spinal circuits for coordinating emotionally related motor behaviors [40], but they may also influence other areas such as the amygdala. Allelic differences in serotonin transporter [5-hydroxytryptamine transporter (5-HTT)] are associated with some of the same socioemotional behaviors that have been linked to the amygdala [41], and social reward in rodents has been shown to be related to the interaction of serotonin with oxytocin in the ventral striatum [42]. Whether such interactions also occur in primates is unknown, although there is evidence that social reward is also related to oxytocin levels in macaques [30].

Such brainstem regions are not often reported in human neuroimaging experiments. The locations of effects were identified with reference to monkey brain atlases [43],[44]. Methodologically, it may be important to note that this region was consistently within our field of view and that our subjects have smaller brains than the humans typically studied in neuroimaging investigations. Second, there is very little head movement in our subjects because they were anaesthetized and held in a stereotaxic frame. Third, we note that because respiration, which disproportionately affects brainstem regions, was artificially maintained at a fixed rate in our anaesethetized subjects, respiratory artefacts could be, and were, removed with 0.1 Hz low-pass filtering.

Not only did the series of analyses confirm the relationship between the six regions and social status, but it was also noticeable that there was not similarly strong or consistent evidence for GM correlations with social status in other brain areas. Only one additional region deserves to be mentioned—the hippocampus and immediately adjacent cortex. We found evidence of a reasonably extensive region of hippocampus in which GM was significantly positively correlated with higher social status in our initial analysis of the left hemisphere, which survived small volume correction for multiple comparisons in the medial temporal ROI. We were able to find evidence of a similar relationship in the right hemisphere. However, we were not able to find evidence for the relationship in our third analysis, which focused on a smaller group of exclusively male subjects at a later date. This region may be especially deserving of attention in future investigations of the neural correlates of dominance. Although neonatal lesions of the hippocampus do not appear to affect social status in macaques [45], the region is smaller in human individuals that have suffered stressful events particularly when they occurred in childhood [46]. Stress has been related to hippocampal degeneration [47], but the relationship between stress and social status is a complex one that may be related to the stability of the social group [48]. There is also evidence that stress decreases as social status increases but that being at the very top of a hierarchy is stressful [33]. Differences in the individuals sampled in the second test or undetected variation in group stability might therefore account for changes in the relationship between hippocampus and dominance in our two measurement periods. However, the relationship between stress and social status was not strong in the present sample (Figures 1–8); metabolites of the stress-related hormone cortisol were not correlated with social status in the current study.

Another social variable, social network size, has previously been shown to be related to brain structure and function in macaques [15],[49]. However, GM in all six of these subcortical regions bore no relationship with the social network size in which animals lived (Figure 9). We were, however, able to identify a second set of regions, the mSTS and the dorsal and rostral PFC, in which GM was related to both social status and social network size (Figure 10).

A complex set of factors determine an individual monkey's social status [50]. Social status in male macaques is not simply the consequence of successful engagement in agonistic behavior but a consequence of success in forming social bonds that promote coalitions between individuals [2]. We suggest that the second set of regions—mSTS and rostral PFC—in which GM was related to both social status and social network size may mediate the way in which dominance is dependent on social bond formation, which is in turn dependent on social cognition. The interactions that mSTS and rostral PFC have with other brain areas while macaques are at rest resemble the interregional interactions of the human temporal parietal junction (TPJ) and rostral medial frontal cortex brain areas, suggesting some relationships between these regions of the macaque and human brain [51]–[54]. In humans, TPJ and rostral medial frontal cortex are associated with the making of inferences about other individuals' actions and intentions [55],[56] and the use of memories of social networks [57]. The PFC region identified in the present investigation of macaques included parts of the principal sulcus just anterior to where neurons have been recorded that are active during the performance of competitive games [25],[58],[59]. Unlike in some other cortical regions, these neurons' activity encoded both choices that the monkey and the competitor made and activity depended on whether the monkey was engaged in a genuinely social situation, playing against another monkey, or against an inanimate computer. Being able to track another individual's behavior as well as one's own would obviously be an advantage in a social setting, and this may be why these areas increase as an individual experiences larger social network sizes; as an individual's social network expands, so too does the number of individuals, and combinations and alliances of individuals, whose behavior must be followed in relation to one's own. A better ability to track others' actions in relation to one's own may, however, also assist an individual in becoming more dominant.

There is some evidence that GM of the macaque amygdala is correlated with an individual's social network size [15]. The relatively restricted region in which such effects have been found was in a more dorsal part of the amygdala than that linked with social status in the current study. However, as explained above, there was no evidence that the amygdala region identified in the current study had any association with social network size (Figure 9). The amygdala is composed of many anatomically dissociable nuclei, each with distinct connectivity, and so it is entirely possible that potentially neighboring nuclei have distinct functions related to different aspects of social life.

In summary, in a series of analyses we have identified brain areas with a consistent relationship with two aspects of dominance. The fact that the association was much stronger in these brain areas than in other brain areas underlines the importance of particular neural processes and argues against all neural processes being equally predictive of dominance. Nevertheless there are a number of other factors that are likely to influence dominance that we were not able to examine in this study. For example, personality types varying in extraversion have been identified in female baboons and have been related to position in the social hierarchy [60].

In humans social hierarchies govern life experiences. Individual social status is correlated with both general and mental health [61]. It is possible that the subcortical areas identified in this study mediate some of these effects. These areas appear to have a relatively simple and direct relationship with social status. By contrast, other cortical regions, mSTS and rostral and dorsal PFC, are associated not just with social status but with other social cognitive processes that are taxed as social network size increases but which are also prerequisites for success in competitive social interactions.

## Methods

The work with animals reported in this study was conducted in accordance with the recommendations of the Weatherall report and under the cover of a license to carry out research with animals issued by the British Home Office.

### Subjects

MRI scans of 25 (3 females) rhesus macaques were used in a DBM analysis of the effect of social status on GM. Data from individuals were used if at least two isotropic 0.5 mm resolution scans were available. Animals were drawn from social groups comprised of five, four, three, and two individuals and five, twelve, one, and seven animals lived in groups of each of these sizes (see Table S3).

The same animals were subsequently also used in a DBM analysis of the effect of social network size on GM, but additional data from other animals were added to this analysis so that the total data set for this second analysis comprised 36 macaques (10 females; see Table S4). The additional animals in this second analysis were ones that could not be included in the dominance status analysis because their dominance status had not been determined. In addition, animals in the second analysis were sometimes drawn from large social groups (groups containing six and seven individuals) that could not be effectively counterbalanced within the dominance status analysis, but of course such larger social group sizes are essential for parametric analysis of social network size.

The relative social status indices used in the first, second, and fourth analyses shown in Figures 1–8 and 10 were adapted from Zumpe and Michael [16] determined by one investigator (J.S.) during a series of 5-minute observations (11 or 12 sessions). Each observation period was preceded by 5 minutes of habituation to the presence of the investigator. The directions of agonistic behaviors (aggressive or submissive) were recorded. Each individual exhibited at least seven single agonistic behaviors during the allotted time period. Behaviors recorded included chasing, escaping, aggressive, and nonspecific social behaviors (for example, resting together). The percentage of dominant interactions out of the total social interactions was calculated to determine cardinal dominance indices. Grooming and mounting are thought to have no necessary relationships with dominance [62] (p100); therefore, these behaviors were classified as nonspecific social behaviors. Both structural MRI and resting-state fMRI data were available for all of 25 animals from this first period of data collection.

For 15 monkeys from the original 25, we were able to collect, after a 4- to 5-month interval, a second structural scan (again composed of at least two isotropic 0.5 mm resolution scans as discussed below; see Table S3) as well as a second independent set of measures of dominance (7 to 10 observation sessions). Measurements were made in the same way as those carried out previously in the first assessment period. This smaller group of animals were all male and came from similar sized social groups. At the time of the first measurement, all animals were living in groups of five or four individuals (5 and 10 animals, respectively). At the time point of the second measurement, 13 of the animals were still living in the same sized social group. Two animals had moved from a group of four to a pair. Although dominance status again appeared stable within the second measurement period, there were some differences between the dominance statuses of individuals recorded at the first and second time periods.

### Hormone Data Collection and Analysis

Fecal samples were collected from a subset of the male animals included in the second social status analysis (13/15). Animals were included when three fecal samples were available (see Table S3). We measured two hormone related metabolites: (1) fecal cortisol metabolite levels [we measured levels of the 3α,11ß-dihydroxy structure cortisol metabolite (3α,11ß-dihydroxy-CM)] and (2) fecal androgen metabolite levels (we also measured epiandrosterone, which reflects testicular function). The samples were analyzed at the German Primate Centre, Leibniz Institute for Primate Research, Goettingen. Analyses were carried out using parametric bivariate analysis in SPSS.

### MRI Data Collection

Protocols for animal care, MRI, and anesthesia were carried out under authority of personal and project licenses in accordance with the UK Animals (Scientific Procedures) Act (1986) using similar procedures to those that we have previously described [15],[63]. During scanning, under veterinary advice, animals were kept under minimum anaesthetic using Isoflurane. A four-channel phased-array coil was used (Windmiller Kolster Scientific, Fresno, CA). Structural scans were acquired using a T1-weighted MP-RAGE sequence (no slice gap, 0.5×0.5×0.5 mm, TR = 2,500 ms, TE = 4.01 ms, 128 slices). Whole-brain BOLD fMRI data were collected for 53 min, 26 s from each animal, using the following parameters: 36 axial slices, in-plane resolution 2×2 mm, slice thickness 2 mm, no slice gap, TR = 2,000 ms, TE = 19 ms, 1,600 volumes. Only structural MRI scans were available from the second data collection period.

### DBM Analysis of Structural MRI Data

Structural MRI data were submitted to a DBM analysis using the Oxford Centre for Functional Magnetic Resonance Imaging (FMRIB) Software Library (FSL) tools FNIRT and Randomise [11],[64]. The logic of the approach is that if a group of brain images can be warped to an identical image, then volumetric changes involved in that warping process give measures of the local differences in brain structure between individuals. Related analyses have previously been described [15].

All the brains were first aligned to the MNI rhesus macaque atlas template [65],[66] using the affine registration tool FLIRT [67],[68], followed by nonlinear registration using FNIRT [69],[70], which uses a b-spline representation of the registration warp field [71]. The resulting images were averaged to create a study-specific template, to which the native GM images were then nonlinearly reregistered. The determinant of the Jacobian of the warp field used on registered partial volumes to correct for local expansion or contraction was extracted—the Jacobian is a matrix of the directional stretches required to register one image to another, and the determinant of this matrix gives a scalar value for the volumetric change implied. The Jacobian values were then used as the dependent variable in the statistical analyses of the effects of social status. The GLM analysis included factors of demeaned social status, age, weight, sex, and the number of structural scans from which each individual's mean structural MRI scan was derived and it was implemented using permutation-based nonparametric testing in the Randomise procedure. We examined both positive and negative contrasts to identify GM regions that were larger in more dominant animals as well as GM structures larger in subordinate animals.

### ROI-Based Approach

We examine the relationship between dominance and GM, after controlling for age, weight, sex, and number of structural scans included in the DBM [15], in a 2,752.5 mm^3^ (22,020 voxels) ROI (ROI mask depicted in Figure 1 in translucent pink) centered over bilateral medial temporal lobe so as to include amygdala. Statistics produced from the FSL Randomise procedure were small volume corrected for multiple comparisons using the threshold free cluster enhancement approach at *p*<0.05 [72].

Next we created a 2,574.5 mm^3^ (20,596 voxels) bilateral ROI (ROI mask depicted in Figure 2 in translucent pink) in the brainstem between the medulla and the midbrain so as to include the location of the serotonergic nuclei. Using the same approach, we performed the FSL Randomise procedure and corrected for small volume multiple comparisons with threshold free cluster enhancement at *p*<0.05.

### Left Hemisphere

In another set of analyses we looked throughout all subcortical regions in the left hemisphere for areas in which effect significance was *p*<0.001 and extended over 10 voxels (corresponding to 1.25 mm^3^). The results of this analysis are shown in Figures 3 and 4.

For illustrative purposes we show the relationships between dominance and the mean Jacobian value extracted from 3.375 mm^3^ mask ROIs placed at the centers of gravity of the regions identified as having a significant relationship with dominance (at *p*<0.005). The Matlab Regstats function was used to calculate the residual DBM effect size and dominance after controlling for confounding effects of age, weight, sex, and number of structural scans that had contributed to the average MRI scan used for each individual.

### Right Hemisphere

We carried out the same analysis in the right hemisphere, again seeking subcortical regions in which effect significance was *p*<0.001 and extended over 10 voxels (corresponding to 1.25 mm^3^). The results of this analysis are shown in Figures 5 and 6. In examining the bilaterality of our effects, we adopt an approach advocated by the originators of MRI voxel-based GM analyses who emphasized that taking into account the spatial extent, across adjacent MRI voxels, of any statistical effect is not necessarily appropriate for GM analyses [11]. The alternative test of robustness involves examining whether effects are bilaterally symmetrical [12]. The premise rests on the assumption that if a statistical effect noted had a chance of occurrence of *p*<0.001 in one brain area under the null hypothesis, then it has the chance of occurring in the same area in both hemispheres with the square of this probability (i.e., *p*<0.00001) [12],[14].

### Second Time Point Analysis

For 15 monkeys from the original 25, we were able to collect a second structural scan as well as a second independent set of measures of dominance. We therefore conducted the same DBM analyses for these 15 animals in both hemispheres. Instead of running whole brain analyses, we now focused on the 12 ROIs (six in each hemisphere) identified in the analysis of data from the first time period. These were AMY (left hemisphere, −10.62, −1.47, −9.85; right hemisphere, 11.21, 0.35, −9.39), PH (left hemisphere, −1.43, −10.02, −5.83; right hemisphere, 2.06, −10.08, −5.49), RN (left hemisphere, −2.763, −22.65, −10.80; right hemisphere, 4.79, −24.46, −12.15), PPUT (left hemisphere, −13.36, −9.87, 1.60; right hemisphere, 13.98, −8.82, 1.06), DS (left hemisphere, −1.99, 2.67, 6.40; right hemisphere, 2.31, 4.27, 5.72), CAUD (left hemisphere, −7.19, −9.03, 9.20; right hemisphere, 7.08, −7.39, 9.22). Note the coordinates refer to the macaque MNI atlas [65]. Each mask was first registered to F99 space and then individually registered from F99 space to each monkey structural scan. The ROI masks were 166.375 mm^3^ in size. We also restricted our analyses to contrasts that reflect the expected direction of effect—that is, positive correlation for AMY, PH, and RN and negative for CAUD, DS, and PPUT. The results of this analysis are shown in Figure 7. Again as in Figures 3–6, for illustrative purposes, we show the relationships between dominance and the mean Jacobian value extracted from 3.375 mm^3^ ROI masks centered over the peak voxel of the significant cluster within the larger volume of interest.

### Social Network Size

We carried out additional analyses to determine whether the regions identified as having a relationship with social status in the first series of analyses were exclusively concerned with social status or whether GM in these regions was also correlated with social network size. To examine whether GM or functional coupling in the regions shown in Figures 1–6 was also significantly correlated with social network size, Jacobian values were extracted from 3.375 mm^3^ ROIs placed on the AMY, PH, RN, PPUT, DS, and CAUD (the same coordinates were used as in the follow-up investigation of dominance using the data collected at the second time point). The residual GM effect size (after age, weight, sex, and number of scans included was controlled) was calculated and correlated with social group size with nonparametric bivariate analyses in SPSS.

A previous report suggested mSTS and rostral and dorsal prefrontal GM correlates with social network size [15]. We therefore overlaid the two statistical images: one showing GM regions with a significant relationship with dominance and one showing GM regions with a significant relationship with social network size after cluster correction using a small ROI mask (*p*<0.05). Specifically, we created anatomically corrected cuboids based on coordinates from [15] where GM correlated with both social status and social network size in the left mSTS and PFC (−25.75, −13.75, −3.25 and 7.25, 21.25, 5.75, respectively). The PFC cuboid in the current analysis was 49 mm^3^, whereas the mSTS was 63 mm^3^. We also replicated the mSTS in the opposite hemisphere by flipping the ROI mask into the other hemisphere. As confirmation we performed a conjunction analysis across the two 3D statistical images by first merging the two images and then determining the lowest *p* value in the overlap cluster.

For illustrative purposes, we again show the relationships between dominance and now also for social group size and the mean Jacobian value extracted from 3.375 mm^3^ ROIs placed at the centers of gravity of the clusters identified by this overlap analysis. The residual DBM effect size of social group size is shown (green) after controlling for confounding effects of social status, age, weight, sex, and number of structural scans that had contributed to the average MRI scan used for each individual. The residual DBM effect size of social status (red) is shown after controlling for confounding effects of social group size, age, weight, sex, and number of structural scans that had contributed to the average MRI scan used for each individual.

### fMRI Analysis of Activity Coupling

Prior to fMRI analysis, the following preprocessing was applied [63]: removal of non-brain voxels, discarding of the first six volumes of each fMRI dataset, 0.1 Hz low-pass filtering to remove respiratory artifacts, motion correction, spatial smoothing (Gaussian 3 mm FWHM kernel), grand-mean intensity normalization of the entire 4D dataset by a single multiplicative factor, high-pass temporal filtering (Gaussian-weighted least-squares straight line fitting, with sigma = 50.0 s). Registration of functional images to the skull-stripped structural MRI scan and to the MNI macaque template [65],[66] was achieved with nonlinear registration using FLIRT [67].

To establish increases in functional connectivity between brain areas as a function of social status, we conducted partial correlation analyses. A 3.375 mm^3^ mask was drawn over the coordinates identified as the centers of gravity of the 12 areas (six in each hemisphere) in which significant DBM effects of social status had been found (AMY, PH, RN, PPUT, DS, and CAUD). This meant that the same coordinates were used both in this analysis and in the follow-up DBM analysis of data collected at a second time point. The masks were then registered to each individual animal's MRI scan and corrected to ensure that they accurately covered the same region in each individual. The individual animal masks were then registered into each individual's fMRI scan space, and the BOLD time series were extracted from each mask in each monkey. We partialled out the confounding influence of the whole brain GM, white matter, and cerebrospinal fluid BOLD time courses by using the FSL general linear model (GLM) tool. To avoid multiple comparisons, we focused on just the left hemisphere—the hemisphere in which we had begun our analysis. Second, in order to further focus our analysis, we examined the correlations in resting state activity in a network of areas with a positive relationship with dominance and a network of areas with a negative relationship to dominance. Therefore, we focused on coupling between the largest area identified as having a positive relationship with dominance (AMY) and each of the other two areas that also had a positive relationship with dominance (PH and RN) and then on the largest area identified as having a negative relationship with dominance (PPUT) and the other two areas that also had a negative relationship with dominance (DS and CAUD).

The time series for four pairs of left hemisphere masks (AMY–RN, AMY–PH, PPUT–CAUD, and PPUT–DS) were then entered into four partial correlation analyses that each controlled for the correlation with the BOLD time series in all 10 other ROIs. So, for example, when examining the partial correlation between left AMY and left RN BOLD times series, we controlled for the correlation with the BOLD time series in left PH, PPUT, CAUD, and DS and right AMY, RN, PH, PPUT, CAUD, and DS. The resulting partial correlation values were then Fisher-transformed and entered into a correlation with individual social status values. This group-level correlation was performed after the variance explained by age, weight, and sex was partialled out using Matlab's Regstats tool. Effectively, at this point we are examining the correlation between each individual's Fisher-transformed value and its social status after controlling for age, weight, and sex.

For illustration we plot the residuals of dominance against the residuals of the partial correlations. We also distinguish which animals had significant regional partial correlation coupling at each end of the dominance spectrum. Those animals where ROI×ROI correlation coefficients were significant at *p*<0.01 and whose dominance scores were less than the group median (31.25) are indicated in blue. In contrast, those animals where ROI×ROI correlation coefficients were significant at *p*<0.01 and whose dominance scores were more than the group median are indicated in red. There is a rough clustering of animals along this dimension suggesting that subordinance is associated with slight but significant positive coupling that becomes a more, slight, but significant anticorrelation the more dominant an animal is. Chi-squared tests in SPSS were used to examine this relationship.

## Supporting Information

Table S1 Six subcortical regions identified in both hemispheres as showing significant correlation with social status.
(DOCX)

Table S2 Correlations between six subcortical regions in both hemispheres and social status at a second time point. An asterisk signifies more than one cluster identified in the volume of interest.
(DOCX)

Table S3 Summary of group housing for animals in the social status analyses. Social status, sex, and group sizes at the time of each scan are shown.
(DOCX)

Table S4 Summary of group housing for animals in the social network size analysis (Figure 10). The group sizes at the time of scanning and sex are also shown. Gray cells correspond to the animals used for a previous study [15]. Not all animals were available for an investigation of social status at a second time point (Table S3). Animal O5 was housed in a group of six when the first MRI scan was taken and the data were used in the analysis of social network size (Table S4). O5 was in a group of two animals approximately a year and a half later when an assessment of social status was first made and a second MRI scan that could be used in the analysis of social status was taken (Table S3).
(DOCX)

## Abbreviations

5-HTT: 5-hydroxytryptamine transporter
ACC: anterior cingulate cortex
AMY: amygdala
BOLD: blood oxygen level–dependent
CAUD: caudate
DBM: deformation-based morphometric
DS: dorsal parts of the septum
fMRI: functional MRI
GLM: general linear model
GM: gray matter
MRI: magnetic resonance imaging
mSTS: mid-superior temporal sulcus
PFC: prefrontal cortex
PH: posterior hypothalamus
PPUT: posterior putamen
RN: raphe nuclei
ROI: region of interest
TPJ: temporal parietal junction

## References

[1] Chiao JY (2010) Neural basis of social status hierarchy across species. Curr Opin Neurobiol 20: 803–809. 20850964 10.1016/j.conb.2010.08.006

[2] Schulke O, Bhagavatula J, Vigilant L, Ostner J (2010) Social bonds enhance reproductive success in male macaques. Curr Biol 20: 2207–2210. 21093261 10.1016/j.cub.2010.10.058

[3] Azzi JC, Sirigu A, Duhamel JR (2012) Modulation of value representation by social context in the primate orbitofrontal cortex. Proc Natl Acad Sci U S A 109: 2126–2131. 22308343 10.1073/pnas.1111715109 PMC3277550

[4] Massen JJ, van den Berg LM, Spruijt BM, Sterck EH (2010) Generous leaders and selfish underdogs: pro-sociality in despotic macaques. PLoS ONE 5: e9734. 20305812 10.1371/journal.pone.0009734 PMC2840023

[5] Klein JT, Shepherd SV, Platt ML (2009) Social attention and the brain. Curr Biol 19: R958–R962. 19889376 10.1016/j.cub.2009.08.010 PMC3387539

[6] Fujii N, Hihara S, Nagasaka Y, Iriki A (2009) Social state representation in prefrontal cortex. Soc Neurosci 4: 73–84. 18633840 10.1080/17470910802046230

[7] Klein JT, Deaner RO, Platt ML (2008) Neural correlates of social target value in macaque parietal cortex. Curr Biol 18: 419–424. 18356054 10.1016/j.cub.2008.02.047 PMC2362498

[8] Raleigh MJ, McGuire MT, Brammer GL, Pollack DB, Yuwiler A (1991) Serotonergic mechanisms promote dominance acquisition in adult male vervet monkeys. Brain Res 559: 181–190. 1794096 10.1016/0006-8993(91)90001-c

[9] Kumaran D, Melo HL, Duzel E (2012) The emergence and representation of knowledge about social and nonsocial hierarchies. Neuron 76: 653–666. 23141075 10.1016/j.neuron.2012.09.035 PMC3580285

[10] Quallo MM, Price CJ, Ueno K, Asamizuya T, Cheng K, et al (2009) Gray and white matter changes associated with tool-use learning in macaque monkeys. Proc Natl Acad Sci U S A 106: 18379–18384. 19820167 10.1073/pnas.0909751106 PMC2759710

[11] Ashburner J, Friston KJ (2000) Voxel-based morphometry—the methods. Neuroimage 11: 805–821. 10860804 10.1006/nimg.2000.0582

[12] Salmond CH, Ashburner J, Vargha-Khadem F, Gadian DG, Friston KJ (2000) Detecting bilateral abnormalities with voxel-based morphometry. Hum Brain Mapp 11: 223–232. 11098800 10.1002/1097-0193(200011)11:3<223::AID-HBM80>3.0.CO;2-F PMC6871793

[13] Watkins KE, Vargha-Khadem F, Ashburner J, Passingham RE, Connelly A, et al (2002) MRI analysis of an inherited speech and language disorder: structural brain abnormalities. Brain 125: 465–478. 11872605 10.1093/brain/awf057

[14] Bridge H, Cowey A, Ragge N, Watkins K (2009) Imaging studies in congenital anophthalmia reveal preservation of brain architecture in ‘visual’ cortex. Brain 132: 3467–3480. 19892766 10.1093/brain/awp279

[15] Sallet J, Mars RB, Noonan MP, Andersson JL, O'Reilly JX, et al (2011) Social network size affects neural circuits in macaques. Science 334: 697–700. 22053054 10.1126/science.1210027

[16] Zumpe D, Michael RP (1986) Dominance index: a simple measure of relative dominance status in primates. American Journal of Primatology 10: 291–300. 10.1002/ajp.1350100402 31979473

[17] Mietchen D, Gaser C (2009) Computational morphometry for detecting changes in brain structure due to development, aging, learning, disease and evolution. Front Neuroinform 3: 25. 19707517 10.3389/neuro.11.025.2009 PMC2729663

[18] Neubert FX, Mars RB, Buch ER, Olivier E, Rushworth MF (2010) Cortical and subcortical interactions during action reprogramming and their related white matter pathways. Proc Natl Acad Sci U S A 107: 13240–13245. 20622155 10.1073/pnas.1000674107 PMC2922153

[19] Buch ER, Mars RB, Boorman ED, Rushworth MF (2010) A network centered on ventral premotor cortex exerts both facilitatory and inhibitory control over primary motor cortex during action reprogramming. J Neurosci 30: 1395–1401. 20107065 10.1523/JNEUROSCI.4882-09.2010 PMC2880444

[20] Buch ER, Johnen VM, Nelissen N, O'Shea J, Rushworth MF (2011) Noninvasive associative plasticity induction in a corticocortical pathway of the human brain. J Neurosci 31: 17669–17679. 22131427 10.1523/JNEUROSCI.1513-11.2011 PMC6623800

[21] Mars RB, Klein MC, Neubert FX, Olivier E, Buch ER, et al (2009) Short-latency influence of medial frontal cortex on primary motor cortex during action selection under conflict. J Neurosci 29: 6926–6931. 19474319 10.1523/JNEUROSCI.1396-09.2009 PMC6665588

[22] Davare M, Rothwell JC, Lemon RN (2010) Causal connectivity between the human anterior intraparietal area and premotor cortex during grasp. Curr Biol 20: 176–181. 20096580 10.1016/j.cub.2009.11.063 PMC2824111

[23] Davare M, Montague K, Olivier E, Rothwell JC, Lemon RN (2009) Ventral premotor to primary motor cortical interactions during object-driven grasp in humans. Cortex 45: 1050–1057. 19345344 10.1016/j.cortex.2009.02.011 PMC2730595

[24] Davare M, Lemon R, Olivier E (2008) Selective modulation of interactions between ventral premotor cortex and primary motor cortex during precision grasping in humans. J Physiol 586: 2735–2742. 18403420 10.1113/jphysiol.2008.152603 PMC2536583

[25] Hosokawa T, Watanabe M (2012) Prefrontal neurons represent winning and losing during competitive video shooting games between monkeys. J Neurosci 32: 7662–7671. 22649245 10.1523/JNEUROSCI.6479-11.2012 PMC6703568

[26] Baez-Mendoza R, Schultz W (2013) The role of the striatum in social behavior. Front Neurosci 7: 233. 24339801 10.3389/fnins.2013.00233 PMC3857563

[27] Santos GS, Nagasaka Y, Fujii N, Nakahara H (2012) Encoding of social state information by neuronal activities in the macaque caudate nucleus. Soc Neurosci 7: 42–58. 21961907 10.1080/17470919.2011.578465

[28] Yoshida K, Saito N, Iriki A, Isoda M (2012) Social error monitoring in macaque frontal cortex. Nat Neurosci 15: 1307–1312. 22864610 10.1038/nn.3180

[29] Yoshida K, Saito N, Iriki A, Isoda M (2011) Representation of others' action by neurons in monkey medial frontal cortex. Curr Biol 21: 249–253. 21256015 10.1016/j.cub.2011.01.004

[30] Chang SW, Barter JW, Ebitz RB, Watson KK, Platt ML (2012) Inhaled oxytocin amplifies both vicarious reinforcement and self reinforcement in rhesus macaques (Macaca mulatta). Proc Natl Acad Sci U S A 109: 959–964. 22215593 10.1073/pnas.1114621109 PMC3271866

[31] Chang SW, Gariepy JF, Platt ML (2012) Neuronal reference frames for social decisions in primate frontal cortex. Nat Neurosci 16 (2) 243–250. 23263442 10.1038/nn.3287 PMC3557617

[32] Gordon TP, Bernstein IS, Rose RM (1978) Social and seasonal influences on testosterone secretion in the male rhesus monkey. Physiol Behav 21: 623–627. 105367 10.1016/0031-9384(78)90140-3

[33] Gesquiere LR, Learn NH, Simao MC, Onyango PO, Alberts SC, et al (2011) Life at the top: rank and stress in wild male baboons. Science 333: 357–360. 21764751 10.1126/science.1207120 PMC3433837

[34] Murray EA, Wise SP, Drevets WC (2011) Localization of dysfunction in major depressive disorder: prefrontal cortex and amygdala. Biol Psychiatry 69: e43–54. 21111403 10.1016/j.biopsych.2010.09.041 PMC3058124

[35] Doya K (2008) Modulators of decision making. Nat Neurosci 11: 410–416. 18368048 10.1038/nn2077

[36] Baez-Mendoza R, Harris CJ, Schultz W (2013) Activity of striatal neurons reflects social action and own reward. Proc Natl Acad Sci U S A 110: 16634–16639. 24062436 10.1073/pnas.1211342110 PMC3799314

[37] Murray EA, Wise SP (2010) Interactions between orbital prefrontal cortex and amygdala: advanced cognition, learned responses and instinctive behaviors. Curr Opin Neurobiol 20: 212–220. 20181474 10.1016/j.conb.2010.02.001 PMC2862864

[38] Izquierdo A, Suda RK, Murray EA (2005) Comparison of the effects of bilateral orbital prefrontal cortex lesions and amygdala lesions on emotional responses in rhesus monkeys. J Neurosci 25: 8534–8542. 16162935 10.1523/JNEUROSCI.1232-05.2005 PMC6725674

[39] Raleigh MJ, Brammer GL, Yuwiler A, Flannery JW, McGuire MT, et al (1980) Serotonergic influences on the social behavior of vervet monkeys (Cercopithecus aethiops sabaeus). Exp Neurol 68: 322–334. 6444893 10.1016/0014-4886(80)90089-8

[40] Kerman IA, Enquist LW, Watson SJ, Yates BJ (2003) Brainstem substrates of sympatho-motor circuitry identified using trans-synaptic tracing with pseudorabies virus recombinants. J Neurosci 23: 4657–4666. 12805305 10.1523/JNEUROSCI.23-11-04657.2003 PMC6740797

[41] Izquierdo A, Newman TK, Higley JD, Murray EA (2007) Genetic modulation of cognitive flexibility and socioemotional behavior in rhesus monkeys. Proc Natl Acad Sci U S A 104: 14128–14133. 17715054 10.1073/pnas.0706583104 PMC1950559

[42] Dolen G, Darvishzadeh A, Huang KW, Malenka RC (2013) Social reward requires coordinated activity of nucleus accumbens oxytocin and serotonin. Nature 501: 179–184. 24025838 10.1038/nature12518 PMC4091761

[43] Saleem K, Logothetis N (2007) A combined MRI and histology atlas of the rhesus monkey brain in stereotaxic coordinates. San Diego, CA: Elsevier/Academic Press.

[44] Paxinos G, Huang X-F, Petrides M, Toga A (2008) The rhesus monkey brain in stereotaxic coordinates. San Diego, CA: Elsevier/Academic Press.

[45] Bauman MD, Toscano JE, Mason WA, Lavenex P, Amaral DG (2006) The expression of social dominance following neonatal lesions of the amygdala or hippocampus in rhesus monkeys (Macaca mulatta). Behav Neurosci 120: 749–760. 16893283 10.1037/0735-7044.120.4.749

[46] Teicher MH, Anderson CM, Polcari A (2012) Childhood maltreatment is associated with reduced volume in the hippocampal subfields CA3, dentate gyrus, and subiculum. Proc Natl Acad Sci U S A 109: E563–E572. 22331913 10.1073/pnas.1115396109 PMC3295326

[47] Uno H, Tarara R, Else JG, Suleman MA, Sapolsky RM (1989) Hippocampal damage associated with prolonged and fatal stress in primates. J Neurosci 9: 1705–1711. 2723746 10.1523/JNEUROSCI.09-05-01705.1989 PMC6569823

[48] Sapolsky RM (1992) Cortisol concentrations and the social significance of rank instability among wild baboons. Psychoneuroendocrinology 17: 701–709. 1287688 10.1016/0306-4530(92)90029-7

[49] Mars RB, Neubert FX, Noonan MP, Sallet J, Toni I, et al (2012) On the relationship between the “default mode network” and the “social brain”. Front Hum Neurosci 6: 189. 22737119 10.3389/fnhum.2012.00189 PMC3380415

[50] Silk J, Cheney D, Seyfarth R (2013) A practical guide to the study of social relationships. Evol Anthropol 22: 213–225. 24166922 10.1002/evan.21367

[51] Sallet J, Mars RB, Noonan MP, Neubert FX, Jbabdi S, et al (2013) The organization of dorsal frontal cortex in humans and macaques. J Neurosci 33: 12255–12274. 23884933 10.1523/JNEUROSCI.5108-12.2013 PMC3744647

[52] Mars RB, Sallet J, Neubert FX, Rushworth MF (2013) Connectivity profiles reveal the relationship between brain areas for social cognition in human and monkey temporoparietal cortex. Proc Natl Acad Sci U S A 110: 10806–10811. 23754406 10.1073/pnas.1302956110 PMC3696774

[53] Rushworth MF, Mars RB, Sallet J (2013) Are there specialized circuits for social cognition and are they unique to humans? Curr Opin Neurobiol 23 (3) 436–442. 23290767 10.1016/j.conb.2012.11.013

[54] Mars RB, Sallet J, Schuffelgen U, Jbabdi S, Toni I, et al (2011) Connectivity-based subdivisions of the human right “temporoparietal junction area”: evidence for different areas participating in different cortical networks. Cereb Cortex 22 (8) 1894–1903. 21955921 10.1093/cercor/bhr268

[55] Behrens TE, Hunt LT, Rushworth MF (2009) The computation of social behavior. Science 324: 1160–1164. 19478175 10.1126/science.1169694

[56] Frith CD, Frith U (2007) Social cognition in humans. Curr Biol 17: R724–R732. 17714666 10.1016/j.cub.2007.05.068

[57] Kumaran D, Maguire EA (2005) The human hippocampus: cognitive maps or relational memory? J Neurosci 25: 7254–7259. 16079407 10.1523/JNEUROSCI.1103-05.2005 PMC6725222

[58] Seo H, Lee D (2008) Cortical mechanisms for reinforcement learning in competitive games. Philos Trans R Soc Lond B Biol Sci 363: 3845–3857. 18829430 10.1098/rstb.2008.0158 PMC2607365

[59] Seo H, Barraclough DJ, Lee D (2007) Dynamic signals related to choices and outcomes in the dorsolateral prefrontal cortex. Cereb Cortex 17 Suppl 1: i110–i117. 17548802 10.1093/cercor/bhm064

[60] Seyfarth RM, Silk JB, Cheney DL (2012) Variation in personality and fitness in wild female baboons. Proc Natl Acad Sci U S A 109: 16980–16985. 23027933 10.1073/pnas.1210780109 PMC3479518

[61] Wilkinson RG (1999) Health, hierarchy, and social anxiety. Ann N Y Acad Sci 896: 48–63. 10681887 10.1111/j.1749-6632.1999.tb08104.x

[62] Fedigan LM (1992) Primates paradigms: sex roles and social bonds. Chicago: University of Chicago Press.

[63] Mars RB, Jbabdi S, Sallet J, O'Reilly JX, Croxson PL, et al (2011) Diffusion-weighted imaging tractography-based parcellation of the human parietal cortex and comparison with human and macaque resting-state functional connectivity. J Neurosci 31: 4087–4100. 21411650 10.1523/JNEUROSCI.5102-10.2011 PMC3091022

[64] Smith SM, Jenkinson M, Woolrich MW, Beckmann CF, Behrens TE, et al (2004) Advances in functional and structural MR image analysis and implementation as FSL. Neuroimage 23 Suppl 1: S208–S219. 15501092 10.1016/j.neuroimage.2004.07.051

[65] Frey S, Pandya DN, Chakravarty MM, Bailey L, Petrides M, et al (2011) An MRI based average macaque monkey stereotaxic atlas and space (MNI monkey space). Neuroimage 55: 1435–1442. 21256229 10.1016/j.neuroimage.2011.01.040

[66] Chakravarty MM, Frey S, Collins DL (2008) Digital atlas of the monkey brain in stereotactic co-ordinates. In: Paxinos G, Huang XF, Petrides M, Toga AW, editors. The rhesus monkey brain in stereotactic coordinates. Amsterdam: Elsevier.

[67] Jenkinson M, Bannister P, Brady M, Smith S (2002) Improved optimization for the robust and accurate linear registration and motion correction of brain images. Neuroimage 17: 825–841. 12377157 10.1016/s1053-8119(02)91132-8

[68] Jenkinson M, Smith S (2001) A global optimisation method for robust affine registration of brain images. Med Image Anal 5: 143–156. 11516708 10.1016/s1361-8415(01)00036-6

[69] Andersson JLR, Jenkinson M, Smith S (2007) Non-linear optimisation. FMRIB Technical Report TR07JA1 TR07JA1.

[70] Andersson JLR, Jenkinson M, Smith S (2007) Non-linear registration, aka Spatial normalisation. FMRIB Technical Report. TR07JA2 TR07JA2.

[71] Rueckert D, Sonoda LI, Hayes C, Hill DL, Leach MO, et al (1999) Nonrigid registration using free-form deformations: application to breast MR images. IEEE Trans Med Imaging 18: 712–721. 10534053 10.1109/42.796284

[72] Smith SM, Nichols TE (2009) Threshold-free cluster enhancement: addressing problems of smoothing, threshold dependence and localisation in cluster inference. Neuroimage 44: 83–98. 18501637 10.1016/j.neuroimage.2008.03.061

