# Supplementary material for: A Neural Circuit Covarying with Social Hierarchy in Macaques
Source: PLoS Biol. 2014 Sep 2;12(9):e1001940. doi: 10.1371/journal.pbio.1001940 (PMC4151964; doi:10.1371/journal.pbio.1001940)
Supplement: Table S1 — Six subcortical regions identified in both hemispheres as showing significant correlation with social status. (DOCX) [file pbio.1001940.s001.docx]

**Supplementary Information.1**

*Supplementary Table1. Six subcortical regions identified in both hemispheres as showing significant correlation with social status.*

| **Region** | **Cluster size (voxels) at p<0.001** | **Cluster size (voxels) at p<0.005** | **Center of gravity of**  **p<0.005 cluster** |
| --- | --- | --- | --- |
| left AMY | 161 | 826 | -10.62, -1.47, -9.85 |
| left CAUD | 23 | 105 | -7.19, -9.03, 9.20 |
| left DS | X | 43 | -1.99, 2.67, 6.40 |
| left PH | 10 | 138 | -1.43, -10.02, -5.83 |
| left PPUT | 213 | 410 | -13.36, -9.87, 1.60 |
| left RN | 84 | 568 | -2.763, -22.65, -10.80 |
| right AMY | 60 | 717 | 11.21, 0.35, -9.39 |
| right CAUD | 7 | 46 | 7.08, -7.39, 9.22 |
| right DS | 94 | 326 | 2.31, 4.27, 5.72 |
| right PH | 21 | 114 | 2.06, -10.08, -5.49 |
| right PPUT | 28 | 126 | 13.98, -8.82, 1.06 |
| right RN | x | 52 | 4.79, -24.46, -12.15 |
