# Supplementary material for: A Neural Circuit Covarying with Social Hierarchy in Macaques
Source: PLoS Biol. 2014 Sep 2;12(9):e1001940. doi: 10.1371/journal.pbio.1001940 (PMC4151964; doi:10.1371/journal.pbio.1001940)
Supplement: Table S2 — Correlations between six subcortical regions in both hemispheres and social status at a second time point. An asterisk signifies more than one cluster identified in the volume of interest. (DOCX) [file pbio.1001940.s002.docx]

*Supplementary Table2. Correlations between six subcortical regions in both hemispheres and social status at a second time point.*

| **Region** | **Cluster size (voxels) showing effect at p<0.05** | **ROI Peak voxel coordinate in anatomical area of interest** | **Peak voxel effect** |
| --- | --- | --- | --- |
| left AMY | 86,7,2* | 12.75, -1.75, -12.25 | 0.009 |
| left CAUD | 106 | 6.25, -8.25, 8.25 | 0.005 |
| left DS | X | X | X |
| left PH | 137 | 1.25, -10.75, -8.25 | 0.002 |
| left PPUT | 29 | 15.25, -11.25, -1.25 | 0.03 |
| left RN | 205 | 3.75, -24.75, -11.25 | 0.011 |
| right AMY | 48,47,15* | -12.25, -1.25, -12.25 | 0.003 |
| right CAUD | 56 | -7.75, -8.25, 9.25 | 0.005 |
| right DS | 8 | -0.75, 1.75, 3.75 | 0.019 |
| right PH | 91 | -0.25, -11.25, 8.25 | 0.015 |
| right PPUT | 183 | -12.75, -9.25, 3.25 | 0.013 |
| right RN | 169,8* | -4.25,-23.75, -9.75 | 0.014 |

* more than one cluster identified in the volume of interest
