# Supplementary material for: A Neural Circuit Covarying with Social Hierarchy in Macaques
Source: PLoS Biol. 2014 Sep 2;12(9):e1001940. doi: 10.1371/journal.pbio.1001940 (PMC4151964; doi:10.1371/journal.pbio.1001940)
Supplement: Table S3 — Summary of group housing for animals in the social status analyses. Social status, sex, and group sizes at the time of each scan are shown. (DOCX) [file pbio.1001940.s003.docx]

*Supplementary Table 3. Summary of group housing for animals in the social status analyses. The group sizes at time of scanning, social status, and employed in analyses are also shown.*

| Subject | Animal | Analysis 1. Animals in group at time of MRI scan | Analysis 2. Animals in group at time of MRI scan | Sex | Analysis 1. Social Status | Analysis 2. Social Status | Hormone analysis |
| --- | --- | --- | --- | --- | --- | --- | --- |
|  |  |  |  |  |  |  |  |
| 1 | O1 | 5 | 5 | M | 81.82 | 100 | x |
| 2 | O2 | 5 | 5 | M | 0 | 0 | x |
| 3 | O3 | 5 | 5 | M | 8.7 | 0 | x |
| 4 | P1 | 5 | 5 | M | 26.09 | 25 | x |
| 5 | P2 | 5 | 5 | M | 36.36 | 27.27 | x |
| 6 | P3 | 4 | 4 | M | 42.11 | 14.29 | x |
| 7 | P4 | 4 | 4 | M | 53.85 | 60 | x |
| 8 | P5 | 4 | 4 | M | 23.08 | 0 | x |
| 9 | P6 | 4 | 4 | M | 57.89 | 50 | x |
| 10 | S1 | 4 | 4 | M | 17.39 | 23.68 | x |
| 11 | R1 | 4 | 4 | M | 33.33 | 63.89 | - |
| 12 | S2 | 4 | 4 | M | 17.39 | 25 | x |
| 13 | S3 | 4 | 4 | M | 0 | 0 | x |
| 14 | S4 | 4 | 2 | M | 60.47 | 0 | - |
| 15 | S5 | 4 | 2 | M | 21.88 | 40 | - |
| 16 | S6 | 4 | 4 | M | 6.67 | - | - |
| 17 | S7 | 4 | 4 | M | 0 | - | - |
| 18 | O4 | 2 | 2 | M | 93.33 | - | - |
| 19 | P7 | 2 | 2 | M | 100 | - | - |
| 20 | P9 | 2 | 2 | F | 59.09 | - | - |
| 21 | P10 | 2 | 2 | F | 31.25 | - | - |
| 22 | O5b | 2 | 2 | F | 0 | - | - |
| 23 | N1 | 2 | 2 | M | 0 | - | - |
| 24 | N2 | 2 | 2 | M | 71.43 | - | - |
| 25 | R2 | 3 | 3 | M | 66.67 | - | x |
