# Supplementary material for: A Neural Circuit Covarying with Social Hierarchy in Macaques
Source: PLoS Biol. 2014 Sep 2;12(9):e1001940. doi: 10.1371/journal.pbio.1001940 (PMC4151964; doi:10.1371/journal.pbio.1001940)
Supplement: Table S4 — Summary of group housing for animals in the social network size analysis (Figure 10). The group sizes at the time of scanning and sex are also shown. Gray cells correspond to the animals used for a previous study [15]. Not all animals were available for an investigation of social status at a second time point (Table S3). Animal O5 was housed in a group of six when the first MRI scan was taken and the data were used in the analysis of social network size (Table S4). O5 was in a group of two animals approximately a year and a half later when an assessment of social status was first made and a second MRI scan that could be used in the analysis of social status was taken (Table S3). (DOCX) [file pbio.1001940.s004.docx]

*Supplementary Table 4 Summary of group housing for animals in the social network analysis (figure 10). The group sizes at time of scanning and sex are also shown.*

| Subject | Animal | Animals in group at time of MRI scan | Sex |
| --- | --- | --- | --- |
|  |  |  |  |
| 1 | O1 | 5 | M |
| 2 | O2 | 5 | M |
| 3 | O3 | 5 | M |
| 4 | P1 | 5 | M |
| 5 | P2 | 5 | M |
| 6 | P3 | 4 | M |
| 7 | P4 | 4 | M |
| 8 | P5 | 4 | M |
| 9 | P6 | 4 | M |
| 10 | S1 | 4 | M |
| 11 | R1 | 4 | M |
| 12 | S2 | 4 | M |
| 13 | S3 | 4 | M |
| 14 | S4 | 4 | M |
| 15 | S5 | 4 | M |
| 16 | S6 | 4 | M |
| 17 | S7 | 4 | M |
| 18 | O4 | 2 | M |
| 19 | P7 | 2 | M |
| 20 | P8 | 6 | M |
| 21 | P9 | 2 | F |
| 22 | P10 | 2 | F |
| 23 | O5 | 6 | F |
| 24 | N1 | 2 | M |
| 25 | N2 | 2 | M |
| 26 | R2 | 3 | M |
| 27 | P11 | 2 | F |
| 28 | O6 | 6 | F |
| 29 | O7 | 7 | F |
| 30 | O8 | 7 | F |
| 31 | P12 | 3 | F |
| 32 | N3 | 1 | F |
| 33 | P13 | 6 | M |
| 34 | P14 | 6 | M |
| 35 | O9 | 2 | F |
| 36 | O10 | 2 | F |

Gray cells correspond to the animals used for a previous study [[15](#_ENREF_15)]. Not all animals were available for an investigation of social status at a second time point (table 3).  Animal O5 was housed in a group of 6 when the first MRI scan was taken and the data were used in the analysis of social network size (table 4).   O5 was in a group of two animals approximately a year and a half later when an assessment of social status was first made and a second MRI scan that could be used in the analysis of social status was taken (table 3).
